# Femicide Circumstances and Harmfulness: Case Report and Focusing Review

**DOI:** 10.3390/diagnostics14131360

**Published:** 2024-06-26

**Authors:** Chiara Stassi, Marco La Mantia, Giuseppe Francesco Lo Re, Valentina Martines, Stefania Zerbo, Giuseppe Davide Albano, Ginevra Malta, Antonina Argo

**Affiliations:** 1 Legal Medicine Section, Department for Health Promotion and Mother-Child Care, Internal Medicine and Medical Specialties (Pro.Mi.SE.), University of Palermo, Via del Vespro 129, 90127 Palermo, Italy; chiara.stassi86@gmail.com (C.S.); marcolm.1993@gmail.com (M.L.M.); stefania.zerbo@unipa.it (S.Z.); giuseppedavide.albano@unipa.it (G.D.A.); ginevra.malta@unipa.it (G.M.); 2 Imaging and Surgical Diagnostics Section, Department of Biomedicine, Neuroscience and Advanced Diagnostics, University of Palermo, Via del Vespro 129, 90127 Palermo, Italy; giuseppe.lore12@gmail.com; 3 Policlinic Hospital Umberto I, Viale del Policlinico 155, 00100 Rome, Italy

**Keywords:** femicide, intimate partner violence, gender-based violence, children, forensic radiology, prevention

## Abstract

(1) Background: Femicide is an increasing phenomenon consisting of the murder of a woman for gender-related reasons. Despite the enactment of new laws aimed at controlling the phenomenon by toughening the penalties and introducing aggravating circumstances, there is an increasing trend that testifies to the persistence of a flaw in the actual measures. (2) Case Presentation. We report the case of the murder of a 32-year-old woman—perpetrated by an ex-husband who refused to accept the end of the marriage—the analysis of which allowed us to frame the case as femicide. (3) Discussion. Despite global awareness of this phenomenon, the identification of risk factors to predict and prevent femicide is of utmost importance. This can be achieved by a multidisciplinary approach involving police officers, legal professionals, hospitals, governmental and nongovernmental organizations, and medico-legal departments aimed at promoting standardized methodologies. (4) Conclusions. We evaluate the contribution of forensic investigations to the identification of key elements that can help frame the murder of a woman as a femicide. Considering the devastating consequences for children who witness this kind of violence within the domestic setting, the planning of more impactful preventive actions is, thus, mandatory to minimize effects on public health.

## 1. Introduction

According to the 1993 United Nations Declaration [1], violence against women is defined as “any act of gender-based violence that results in, or is likely to result in, physical, sexual, or psychological harm or suffering to women, including threats of such acts, coercion or arbitrary deprivation of liberty, whether occurring in public or in private life”. It is estimated that this phenomenon affects approximately 35.6% of women worldwide and is mainly perpetrated by an intimate partner (30% of cases), whether current or former (the so-called “intimate partner violence”) [2,3,4,5,6,7,8,9,10].

Intimate partner violence represents a serious social and public health issue worldwide, which, also due to the impact of mass media, continues to gain attention not only for its increasing incidence and severity but also for its related escalation to murder, the latter accounting for approximately 38.6% of women killed by their (ex)partner [6,7,8,9]. According to Sorrentino et al. [2], in Italy, 31.5% of women aged 16 to 70 years suffer psychological and/or physical and/or sexual violence, which is perpetrated by the current partner in 26.4% of cases and by former partners in 46.1% of cases.

In such a context of gender-based violence, the gender-related killing of women and girls, also referred to as “femicide”, has emerged. Although a precise and unique definition is still lacking, femicide can certainly be intended as the murder of a woman just because she is a woman; as a matter of fact, all possible forms of femicide, mostly intimate partner violence but also misogynist-based slaying, “honor” murder, and sexual murder, share the same ground: the supposed position of inferiority of a woman compared to that of a man [9,10].

The increasing awareness of this phenomenon has led the Italian Parliament to introduce new laws and promote measures aimed at providing protection for women and, thus, at preventing femicide since 2019; nonetheless, the increasing number of such crimes testifies to a flaw in such preventive strategies. Possible explanations might lie in incomplete knowledge of the real extension, mechanisms, and evolution of the phenomenon due not only to the lack of standardized methodologies but also to missing/fragmented information from police officers, legal professionals, hospitals, governmental and nongovernmental organizations, and medico-legal departments [10,11]. This context leads to the consideration of femicide as a preventable death that should be treated as a social and public health problem and a distinct form of homicide in the legal code.

In this regard, we present the case of a 32-year-old woman with murder who, based on both circumstantial data and autopsy findings, was diagnosed with femicide according to the actual criteria in the current literature.

## 2. Case Presentation

Following a spontaneous report by the ex-husband to the police, a 32-year-old woman was recovered in her house’s bathroom, lying on the floor and concealed by a quilt. Once the quilt was removed, the corpse appeared covered in blood, and multiple sharp force wounds were detected all over the body; the bathroom walls and fixtures were all spattered in blood as well.

The corpse was, thus, transferred to the mortuary, where a preliminary *post mortem* CT (PM-CT) was performed. The main findings consisted of several sharp force wounds, either shallow or penetrative, spread throughout the body, the most severe of which compromised the underlying structures at different levels: at the face level, nasal and left zygomatic bone fractures, together with right eye bulb deformation; at the neck level, C_2_ left transverse process fracture; at the thoracic level, fractures of the posterior arch of the II left rib and of the anterior arches of III and IV bilateral ribs, with associated trespassing lacerations of the heart, bilateral pneumothorax, and hemothorax.

**Figure 1:**
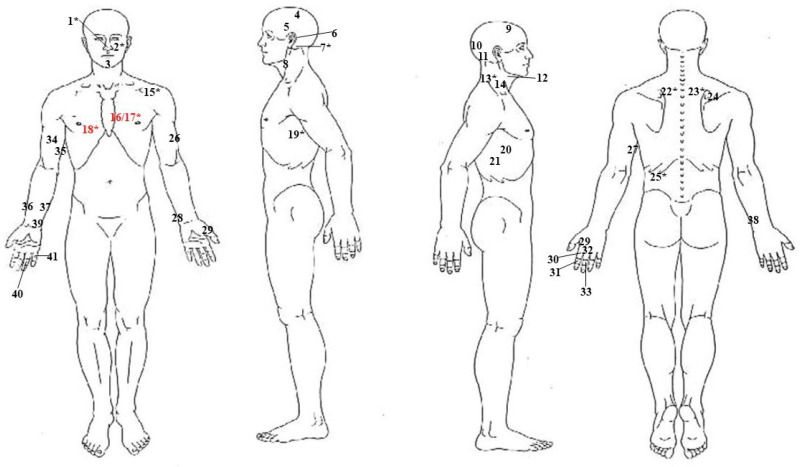
Overview of the sharp force wounds inflicted on the victim. The * symbol indicates penetrative lesions; lesions identified as lethal lesions are highlighted in red.

Upon external inspection, a total of 41 sharp force wounds were detected, which were variably spread throughout the face, head, neck, thorax, back, and both upper limbs; seven wrist/hand injuries were assessed as typical defense injuries (Figure 1). In addition to sharp force wounds, very slight ecchymosis was also detected in the neck region.

**Figure 2:**
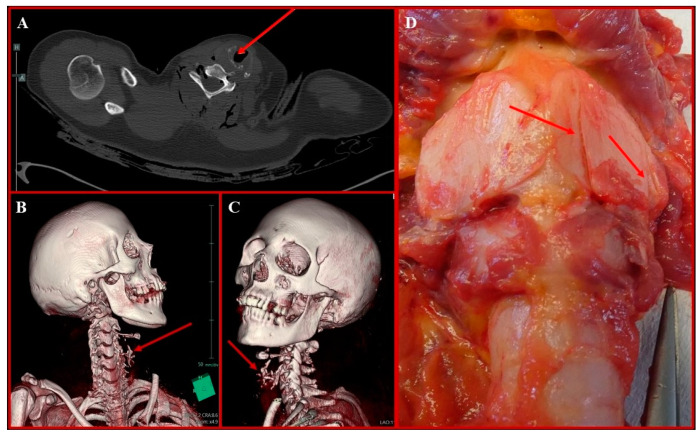
(**A**) Axial PMCT image showing extensive right paramedian thyroid cartilage fragmentation, further highlighted on 3D coronal reconstruction (**B**,**C**) and a cadaveric section (**D**) (red arrows).

The study of the internal paths during the cadaveric inspection allowed the identification of 12/41 penetrative lesions (4 localized on the head–neck district; 5 localized on the thorax; 3 localized on the back), all of which corresponded to the fractures and internal organ lacerations previously detected on PM-CT. Specifically, both lungs appeared collapsed and pale, with evidence of hemorrhagic contusions in areas III and IV of the right ribs; both pleural spaces were filled with blood. The left lung presented a trespassing continuous solution at the basal lobe; on the right lung, trespassing continuous solutions were detected at the base of the median lobe and at the basal lobe. The pericardium had two continuous solutions on its anterior surface, which were in direct continuity with two trespassing continuous solutions on the anterior surface of the left atrium and on the posterior surface of the left ventricle; such lacerations appeared to be reunited in one large continuous solution detected on the posterior surface of the pericardium. In addition to these findings, a few continuous solutions were also detected on the thyroid cartilage (Figure 2).

**Figure 3:**
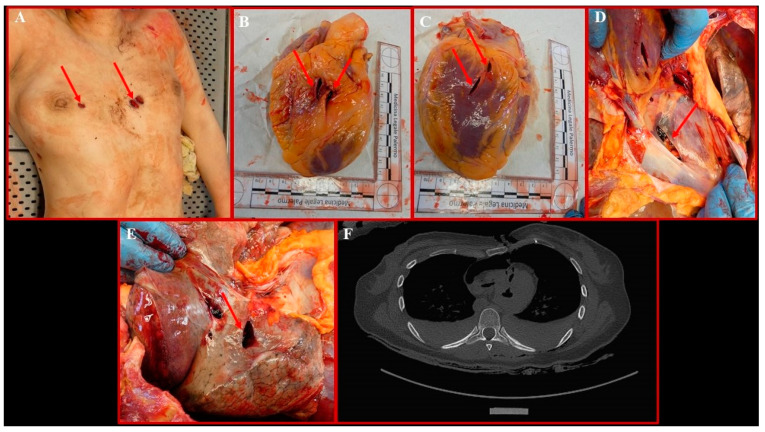
(**A**) Details of the sharp force-induced wounds identified as lethal. The stabs on the left hemithorax are in continuity with two trespassing lacerations of the heart on the anterior surface of the left atrium and the posterior surface of the left ventricle (**B**,**C**). On the posterior surface of the pericardium, the two paths overlapped in a unique path (**D**). (**E**) Detail of the trespassing laceration at the basal lobe of the right lung, which is in continuity with the stab on the right hemithorax. (**F**) Axial PMCT image showing bilateral pneumothorax and hemothorax generated because of lethal lesions; trespassing lacerations of the heart can also be observed.

Three out of the twelve penetrative lesions localized on the thorax were ultimately considered lethal (Figure 3A). The path of the lesions identified with the numbers 16 and 17 (Figure 1), both localized on the left side of the sternum, a few mm apart, at the III intercostal space level, is represented by a first laceration of the III and IV chondro-sternal joints, followed, in sequence, by lacerations of the anterior surface of the pericardium, the anterior surface of the left atrium, and the posterior surface of the left ventricle (Figure 3B,C). On the posterior surface of the pericardium, the two paths, very close to one another, overlapped in a unique path (Figure 3D), which trespassed the left lung basal lobe, finally ending at the X left intercostal space on the posterior surface of the ribcage. The third lethal lesion, identified as number 18 (Figure 1), was localized on the right side of the sternum at the III intercostal space level as well; in this case, the III and IV chondro-sternal joints were first lacerated, followed, in sequence, by lacerations of the median and basal lobe of the right lung (Figure 3E), both trespassed, and of the IX intercostal space, finally ending on the D_10_ body and right transverse process.

The extensive damage to the heart and lungs was responsible for bilateral pneumothorax and hemothorax, as highlighted by the PMCT images (Figure 3F).

Toxicological investigations on the body were negative for any tested abuse and/or alcoholic substance. Since the trousers of the night robe and slips were lowered, further investigations were carried out to establish whether there had been sexual abuse, which resulted in negative results. This is in line with what was reported by the ex-husband, who said that when he entered the bathroom to assault the ex-wife, she was sitting on the toilet.

For the reasons leading to homicide, the man reported that the woman had broken up with him a few years earlier because she was no longer in love with him; since he could not accept such rejection due to the feeling of frustration, at first, he had repeatedly approached her to start over, even though a restraining order had been issued by the police. When the woman later admitted she had another partner, he pretended to have resigned and, to maintain access to the house, asked the woman for permission to come by sometimes to visit their three children. The morning of the homicide, while the two little kids were at the grandparents and the older one was still sleeping in his room, he came by. As a quarrel was about to start, the woman ended the dialog, stating that she would not leave her new partner, and went to the bathroom. The man, thus, took a knife, entered the bathroom, grasped the woman’s throat so that she could not scream, and repeatedly stabbed her. Once the woman died, he took his clothes off and put on some clean ones; then, he covered the corpse with a quilt, awakened the older son, and took him to the grandparents. After preparing a small rucksack, he then went to the police to confess the fact and guided the officers to the recovery of the knife used: a 20 cm point-cut blade, stained in blood upon recovery, which was considered compatible with the inflicted wounds.

When questioned, the sister and a friend of the victim said that the woman had recently confessed to them that she was scared since the ex-husband had threatened her of death a few times had she not left the actual partner, adding that he was not scared of prison. The sister also reported that the 14-year-old son was constantly worried about his mother’s safety, so that he would not leave her alone when the father was present; as the mother died, he started suffering from posttraumatic stress disorder and panic attacks, together with a constant guilty conscience since he kept thinking that, had he woken up earlier that morning, he could have protected his mother, who would still be alive.

## 3. Discussion

Although femicide can generally be intended as the extreme apex of a continuum of violence against women, a global consensus on a precise definition is still lacking. This leads to major limitations concerning the identification of the key elements that differentiate femicide from other generic terms often used as synonyms (e.g., murder, homicide, manslaughter, crimes of passion), thus affecting the possibility of elaborating reliable statistical data and, therefore, adequate preventive strategies [8,11,12].

In the attempt to overcome such limitations, several authors have tried to better frame the phenomenon by analyzing, within a forensic context, the factors that could represent a risk for the escalation to femicide [2,6,7,8,9,10,11,12,13,14].

The main risk factors relate to the aggressor, type of relationship, and motive. In line with the case reported, the aggressor is typically a known person, mostly a partner or ex-partner with whom the victim shared an abusive relationship characterized by continuous physical and/or psychological aggressions and death threats. The motive is usually represented by extreme jealousy due to an imaginary or real assumption that the victim is dating some other man, which frequently leads to so-called “honor” killing, or by a sense of ownership leading the aggressor to refuse the advocated end of the relationship. In this scenario, as explained by Garcia-Vergara et al. [9], the lethal act is justified by the distorted conceptions of the aggressor about authority, possessiveness, and the subordinate position of women, which result in the perception of a loss of control of the partner through separation or divorce.

A related risk factor for femicide, thoroughly analyzed by McFarlane et al. [15] and to which separated/divorced women are mostly exposed, is stalking behavior, described as the act of the perpetrator following or spying on the victim or trying to keep in touch with her against her will. Consequently, positive criminal records for arrests and/or restraining orders following offenses exerted against women can be enlisted, as can risk factors for femicide [8,16]. These data are in line with the history of stalking reported in the present case, in which a restraining order for the former husband was issued by the police.

As in the present case, in which the woman was killed in her home using a knife, femicides are most likely carried out within an indoor setting, where pointed or edged tools (first knives) are easily available. This is in accordance with the main findings in the literature, in which sharp force injuries and gunshot injuries are reported as the main causes of death in femicides in Italy, followed by suffocation/strangulation and blunt force traumas [6,7,8,9,10,11,12,13]. In their work, Biehler-Gomez et al. [13] further evaluated injuries in relation to the age of the victims, observing that victims less than 50 years old mostly died from sharp force injuries and asphyxia, whereas those more than 50 years old were mostly killed by blunt force traumas. They also related such trends to the better physical capacity of younger women to defend themselves, thus inducing aggressors to use more aggressive tools, such as sharp weapons. Such a statement is consistent with the frequent evidence of defense injuries in younger victims, as in the present case. The psychological element of the crime is fundamental in the Italian legal system for the definition of the specific type of crime and any aggravating circumstances, as well as for the definition of the punishment. In the case of femicide, it is essential to define the crime. Forensic analysis often helps investigations in defining the psychological element of the crime, identifying the lethal blow compared to other injuries and characterizing the injuries, and any signs of active defense by the victim. In the case presented, the attacker confessed to the murder.

However, to extrapolate some characteristics that help to suspect, during an autopsy of a female subject, that it was a femicide, we can identify from the specific case the following elements: the high energy of the lesions and the homicidal will (it is not a demonstrative aggression resulting in death); the multiplicity of lesions; and the absence of disfigurement of the body (no firearms, no mutilations).

Despite these efforts, the extent of the problem does not seem to be contained. According to ISTAT (Italian National Institute of Statistics), in Italy, a total of 303 murders were registered in 2021—the year of the present femicide—139 of which (45.9%) had been perpetrated within a relationship or familial context; 100 victims were women, 58.8% of whom were killed by a partner or ex-partner, with an increasing trend compared to 2020 statistics (57.8% in 2020) [17]. According to the United Nations Office on Drugs and Crime (UNODC), almost 29% of female homicides in Europe can be attributed to crimes intentionally committed by an intimate partner [18].

Due to such high numbers, several documents and laws have been enacted over the years [6,10,12,18,19]. In Italy, femicide was first addressed with the introduction of Law n. 119/2013 and the ratification of the Istanbul Convention in the same year, in which aggravating circumstances and penalties for gender-based crimes were revised [20]. In 2019, the so-called “Red Code” Law [21] introduced new crimes, including stalking behavior, further toughening the related penalties. New changes in the Italian Penal Code were also introduced in June 2023, following a femicide perpetrated at the expense of a pregnant woman by her former partner. Specifically, several measures have been implemented with the aim of accelerating the judicial process, while other precautionary measures have been strengthened, such as the appliance of the electronic bracelet and distance limits set at 500 m either from the victim’s home or the places she usually visits.

According to the Italian Criminal Law, except for female genital mutilation, the gender of a person suffering a crime does not have relevance; as a matter of fact, the Italian legal system does not provide measures aimed at specifically and exclusively fighting violent behaviors exerted against women. Nonetheless, the implementations have provided specific aggravating circumstances for life sentences, namely, if the murder involves sexual violence or is committed to a relative (ascendant or descendant), partner (even if legally separated or divorced), or former partner (regardless of whether the perpetrator lived with the victim). In line with this, the ex-husband of the victim in the case reported here has been sentenced to life imprisonment following the recognition, as aggravating circumstances, of murder of the ex-wife and cruelty for acting on a person in a state of poor defense capability (the victim was sitting in the bathroom when the husband unleashed his “homicidal fury”), which led to 41 injuries.

Another issue worth mentioning is the so-called “*witnessed violence*”, defined by the CISMAI (Italian Coordination of Services against Child Mistreatment and Abuse) as “*the child’s experience of any form of ill-treatment, carried out through acts of physical, oral, psychological, sexual and economic violence on reference point figures or other affectively significant adult and minor figures*”. The importance of this phenomenon lies in the emotional, behavioral, and cognitive consequences for children; thus, it has evolved into a serious sociosanitary problem [22]. In fact, children of abused women have a greater risk of developing anxiety, depression, or somatic complaints [23]. In the worst cases, having witnessed violence within the family during childhood can induce the reproduction of the same patterns of violence in adulthood [8]. For this reason, witnessed violence can also be considered a potential risk factor for femicide [24].

## 4. Conclusions

Despite the enactment of several laws and the toughening of penalties, femicide still represents an increasing phenomenon in Europe, considering a unique, globally accepted definition [6,25]. In this context, forensic investigations can provide valuable help by identifying femicide patterns that could be useful for the identification of risk factors and, thus, for better planning preventive measures [6,7]. Much effort has yet to be done from a juridical point of view since several measures do not seem to be enough to guarantee protection; for instance, although stalking behavior counts among crimes, it does not allow the release of a restraining order unless it is associated with physical assault [25]. The need for effective preventive measures should also consider the devastating effects of witnessed violence by children trauma, not only for the related psychological problems it could cause but also for the possible risk of increasing the crime of femicide due to the desire to reproduce in adulthood and the violence experienced during childhood within the family in the form of offender behavior [26,27,28]. A reduction in the crime of femicide by evidence-based strategies within current data obtained from the literature is yet to be obtained in all countries [29,30]. More efforts by politics on surveillance and research are needed, with deep knowledge of both sociodemographic and situational factors and victims and aggressors, to lead effective prevention measures.

## Data Availability

Data are available on request to the corresponding author.
